# Deep Features Homography Transformation Fusion Network—A Universal Foreground Segmentation Algorithm for PTZ Cameras and a Comparative Study

**DOI:** 10.3390/s20123420

**Published:** 2020-06-17

**Authors:** Ye Tao, Zhihao Ling

**Affiliations:** Key Laboratory of Advanced Control and Optimization for Chemical Processes, Ministry of Education, East China University of Science and Technology, Shanghai 200237, China; taoye19920606@126.com

**Keywords:** moving object segmentation, PTZ camera, convolutional neural network, image alignment

## Abstract

The foreground segmentation method is a crucial first step for many video analysis methods such as action recognition and object tracking. In the past five years, convolutional neural network based foreground segmentation methods have made a great breakthrough. However, most of them pay more attention to stationary cameras and have constrained performance on the pan–tilt–zoom (PTZ) cameras. In this paper, an end-to-end deep features homography transformation and fusion network based foreground segmentation method (HTFnetSeg) is proposed for surveillance videos recorded by PTZ cameras. In the kernel of HTFnetSeg, there is the combination of an unsupervised semantic attention homography estimation network (SAHnet) for frames alignment and a spatial transformed deep features fusion network (STDFFnet) for segmentation. The semantic attention mask in SAHnet reinforces the network to focus on background alignment by reducing the noise that comes from the foreground. STDFFnet is designed to reuse the deep features extracted during the semantic attention mask generation step by aligning the features rather than only the frames, with a spatial transformation technique in order to reduce the algorithm complexity. Additionally, a conservative strategy is proposed for the motion map based post-processing step to further reduce the false positives that are brought by semantic noise. The experiments on both CDnet2014 and Lasiesta show that our method outperforms many state-of-the-art methods, quantitively and qualitatively.

## 1. Introduction

Foreground segmentation is an activate research topic in computer vision [1], as it is a stepping stone for video surveillance and many video analysis methods by extracting useful information from videos. During the process of foreground segmentation, moving and informative objects are separated from the static or periodic moving background objects (e.g., road or waving water), which is the first step for many hot applications, such as action recognition [2], clothing recolouring [3], and intelligent transportation system [4].

Traditionally, the foreground segmentation methods are designed under the assumption that cameras are stationary. However, the field of view (FOV) of the stationary camera is limited by its fixed location. The multi-cameras systems are adopted for video surveillance and object tracking tasks to overcome this limitation, because it can cover different angles and shots. Despite these advantages, the multi-camera also brought challenge issues, such as installation cost, multi-camera collaboration, and object re-identity. In contrast, the PTZ camera can both avoid the issue brought by multi-cameras and take the advantages of the broad vision field and focusing the region of interest with a high resolution. Therefore, in the last few years, PTZ cameras began to be widely used in many surveillance circumstances. Meanwhile, the background change brought by the panning, tilting and zooming of cameras also raises the challenge, named PTZ camera challenge, to foreground segmentation methods.

Under this circumstance, many efforts are made to extend the proven methods from stationary cameras to PTZ cameras. For example, the sample-based background subtraction method PAWCS [5] shows its robustness to moving cameras by storing the various background representations as background words. Instead of changing the inner mechanism, the panoramic background image [6] enables the traditional methods to be directly used for PTZ cameras by simply registering the current frame to the background model. In order to better adapt to the moving camera, some inner mechanisms are also designed to combine with the panoramic background. In [7], keypoints are not only used for feature matching, a key step for panoramic image generation, but also used for clustering prepared for foreground detection and also a spatio-temporal tracking of keypoints are attached to distinguish the background. Similar to the panoramic-based method, the motion compensation techniques are also a good choice for moving camera foreground segmentation methods, in which the current image is also registered with the background model, but the background model is not an extended image as a panorama, so this decreases the computation time and the memory allocation [8]. The recent method [9] further reduces the computation load by grid-based keypoints and factors proposed for static camera background subtraction, such as local pixel difference and Gaussian filter, are also used for finer performance. Be contrast to the thought dealing with the PTZ camera challenge by frames alignment, the motion segmentation based methods [10,11] are proposed based on the dense optical flow or motion trajectories. The optical flow estimation based method [10] proposed a constrained RANSAC algorithm to distinguish the background optical flow field in order to extract the foreground mask. The motion segmentation is further fused with appearance-based foreground estimation in [11], where an innovative Mega-pixel denoising process acts as a key part in this fusion by smoothing the probability estimates while using colour segmentation. Comparing with compensation-based methods, they do not rely on explicit camera motion models but they are usually based on the two assumptions that the majority of the pixels in a scene belongs to the background, and foreground objects and camera motion differ in terms of motion pattern.

When comparing with the traditional moving object segmentation method, the convolutional neural network (CNN) based methods show a great advantage in terms of performance, which benefits from the data-driven feature extractor and classifier. Even on the PTZ camera challenge, some CNN-based methods [12,13] still get far higher F-Measure on the PTZ category of CDnet2014 dataset than other traditional methods. However, as these CNN-based methods are supervised, they face the trade-off between accuracy and generalization ability. For example, the performance of FgSegNet [12] drops greatly on unseen videos, although it reaches human-level performance on seen videos. For one method, the unseen videos are the videos whose background and foreground objects have never used in the setup stage of the method. To pursue the generalization ability, the universal DFFnetSeg [14] is proposed for the sudden scene change challenge, whereas it is not robust to the continuous scene change situation which is common in PTZ camera video.

Inspired by the unsupervised homography estimator model [15], we propose a deep features homography transformation and fusion network for foreground segmentation (HTFnetSeg) to tackle the PTZ camera challenge, which can both offset the camera motion by homography transformation and take the advantage of the generality of DFFnetSeg. In detail, the differentiable Tensor Direct Linear Transform (TDLT) layer and Spatial Transformation (ST) layer proposed in [15] enable the homography estimator to be trained in an unsupervised manner, which avoids the demand for the time-consuming manual ground truth labelling. In many cases, the homography ground truth is not available from the surveillance videos, so the unsupervised manner also extends the usage field of this homography estimator. However, when the most parts in the centre region of one image are covered by the foreground (which is common in surveillance videos), the network in [15] might align the image based on the foreground motion instead of the background motion, since it crops the centre regions of two images as its input patches. As our deep features homography transformation and fusion network (HTFnet) aims to align the deep feature inputs of fusion network (FusionNet) to adapt it to the moving surveillance camera challenge, the homography estimation part of it is demanded to be robust to the occlusion of large foreground objects. Therefore, a semantic attention mask, based on a semantic segmentation network PSPNet, is proposed to pre-process the input pair and modify the loss function of the homography regression model. The input pair in our homography estimator consists of two original frames without cropping, so, to further deal with the black boundary of the warped image, the masked loss function combining with a constraint element is proposed, which enables the input pair to well replace the cropping strategy in [15] with the benefit of a bigger receipt field. The deep features generated during the semantic attention mask generation step are reused inside the spatial transformed deep features fusion network (STDFFnet), which aligns the deep features by spatial transformation layer to predict the foreground mask by the FusionNet, in order to reduce the computing complexity. Finally, the key post-processing step designed for semantic noise restraint in DFFnetSeg method is modified to adapt to the PTZ camera challenge. Specifically, a relatively conservative strategy for region-based motion map generation is proposed comparing with the original version proposed in [14], as the alignment deviation exists in the aligned images. This deviation could be overcome during the FusionNet stage, but, during the post-process stage, the greedy motion map that is proposed in DFFnetSeg is too sensitive to the deviation, because it just activates one location depending on a very weak hint of motion. Therefore, in HTFnetSeg, the motion map is modified to demand a stronger hint to activate its pixel location to tackle the noise brought by the alignment deviation.

The contributions of this paper are three folds:

- A semantic attention based homography estimator is proposed to reduce the foreground object noise to the image alignment. The semantic attention masked inputs enable the regression network to receive fewer foreground objects than original images and the masked loss function is also combined to focus on backpropagating the gradient from the “background region” defined by the semantic prediction. Two additional elements are also proposed for the loss function to enhance the model to converge stably and evenly during training.
- A spatial transformed deep features fusion network (STDFFnet) is proposed to estimate the foreground mask based on features comparison. It warps the deep features as the input of FusionNet instead of just warping the images as the input of the feature extractor in DFFnet. This deep features warping strategy can reduce the calculation load of our method by enabling the reuse of the features generated during homography estimation stage.
- A conservative strategy for region-based motion map generation is proposed to enable our post-processing step to be robust to the camera motion by demanding the stronger motion hint than the corresponding in DFFnetSeg method. The region-based motion map generation step acts as the post-processing step to reduce the false positives that are caused by the semantic noise.

The paper is structured, as follows. In Section 2, the related works are discussed. In Section 3, the HTFnetSeg method is presented in detail. In Section 4, the experimental settings including the implementation detail of parameters training and hyper-parameters settings are introduced. In Section 5, the results of our method on two public datasets are compared with state-of-the-art methods quantitively and qualitatively. In Section 6, the relevant inferences that are obtained from the development are discussed. In Section 7, the HTFnetSeg method is concluded and the future work is discussed.

## 2. Related Work

These years, a trend shows that the attention of surveillance video analysis tasks is moving from the stationary cameras to moving cameras such as widely used PTZ cameras. The foreground segmentation method also follows this trend. Besides, the convolutional neural network based methods that take advantage of data-driven feature extraction have shown great success in computer vision tasks. Therefore, since 2016, increasing CNN-based background subtraction methods have been proposed and have shown a great breakthrough in this field. However, similar to the trend of traditional methods, more efforts of deep learning methods tend to be paid for the moving camera challenge these days.

In terms of the traditional methods, the foreground extraction methods that are designed for stationary cameras commonly consist of three main parts: background model design and initialization, the comparison between the current frame and background model, and background model maintenance. The competitive methods include Gaussian mixture model (GMM) based [16], sample-based [5], and codebook based [17] methods. They are designed to tackle different traditional challenges such as hard shadow, illumination change, dynamic background, and camera jitter, but their applications are limited by the assumption that the camera is stationary. Among them, many sample-based methods normally keep about 50 different samples as the background model, which enables them to be robust to the background changing in a certain range, and as in PAWCS [5], a feedback control scheme is proposed based on the background dynamics, which further makes PAWCS robust to background change, especially when the background in the background model appears in the current frame again. SWCD [18] tried to deal with the PTZ camera challenge by the background model updating strategy who detects the scene change based on histogram equalization and Sobel operator. In addition to the background maintenance strategies of methods that may be robust to the PTZ camera challenge to some degree, the panoramic image construction technique is widely used [6,19,20], because, after generating the panoramic image and registering the observed frames, the static scene background subtraction methods can be directly modified to the PTZ camera background subtraction. For example, [20] constructs a panoramic frame by SIFT features [21] and Random Sample Consensus (RANSAC) [22] technique before modelling it by Gaussian probability density function, and the foreground is detected after registering the observed frames to its panoramic Gaussian mixture model (PGMM). However, panorama-based approaches tend to suffer from the error accumulation that is caused by stitching error. Besides, when the field of view (FOV) of a PTZ camera is large, a considerable amount of memory is needed for storing the big panoramic image and the searching space of feature matching for image registration is also large. To overcome this drawback, a compensation-based approach is proposed for moving cameras by finding a transformation matrix that indicates the displacements of two consecutive frames that are caused by the camera movement. Typically, the scene conditional background update moving object detection method (SCBU) [23] defines three scene condition variables: background motion, foreground motion and illumination changes as the evidence for background warping and update, respectively, in which the Kanade–Lucas–Tomasi Feature Tracker (KLT) and RANSAC are used for detecting and matching motion. In addition to the background model based methods that compare the reference frame with the current frame, the trajectory-based methods [24] are proposed on the trajectories over up to *t* frames. After the trajectory clustering, MLBS [24] uses the Kernal density Estimation (KDE) method on the appearance model to infer the probability map and generate the final pixel-wise segmentation based on Graph-cut technique.

Recently, the CNN-based methods are widely researched because of their high-quality performance and data-driven features. The first CNN-based background subtraction method [25] was proposed in 2016, which provided a new frames comparison method based on a convolutional neural network whose architecture is similar to LeNet-5. Based on this work, many deep learning moving object segmentation methods spring out and surpass the traditional methods with a great gap (around 20% in terms of F-Measure), as shown on the benchmark website provided by CDnet2014 [26]. However, the background model based methods, like [25,27], show a great drawback on PTZ camera challenge, because even with a powerful classifier, the networks are still hard to get a good result without the matched reference frame or background model. To avoid the reference frame matching problem, the foreground segmentation methods [12,13] are designed to segment the foreground object based only on a single frame other than the comparison, which obtains high-quality results and is robust to various challenges. For example, FgSegNet [12] and its variants occupy first several entries on CDnet2014 with F-Measure around 0.98, even in the PTZ camera challenge where the F-Measure of DeepBS [27] drops by 43% when compared with its average performance over 11 different challenges. FgSegNet is constructed in an encoder-decoder manner, with triplet VGG-16 as the encoder and a transposed CNN as the decoder, but its good performance highly relies on the training data: 200 frames selected randomly from the same videos as test videos by focusing more on the frames that contain some foreground objects. It means when FgSegNet is tried to be implemented on an installed camera, the frames with foreground objects are demanded to be captured and labelled in pixel-wise as training data to achieve its human-level performance. However, this condition cannot always be fulfilled. To avoid the demand for the pixel-wise label from unseen videos, a reconstruction-based CNN method [28] is proposed to construct the reference image patch by the image-completion network (ICNET), which can fill in the masked centre region of the input patch by reconstruction. The change detection network (CDNET) in [28] is a general model for comparing the difference between the input patches, whereas when ICNET-CDNET needs to be applied to a new scene, the ICNET needs to be trained on the background patches from the new scene to get proper performance. When comparing with FgSegNet, the advantage of background patches demanded in [28] is that the background frames are relatively easier to pick than the pixel-wise label. However, the demand for background patches indicates that the ICNET is still not general enough.

To deal with the dependence of supervised methods on the training data, transferring the pre-trained model from other tasks can enhance the generality ability of CNNs. The semantic features extracted by the pre-trained PSPNet [29] are successfully used as the evidence for comparing, as shown in DFFnetSeg [14]. With the help of the deep features trained on the semantic segmentation task, the fusion network shows its generality to the unseen videos and robustness to the dynamic noise and ghost problem. DFFnetSeg can also adapt to the sudden scene change fastly by combining with a scene change detector based background update strategy, whereas this strategy is not enough for the continuous scene change and PTZ camera challenge. Similar to the solution for the PTZ camera challenge in traditional methods, the reference frame alignment based foreground segmentation methods are proposed. A CNN-based change detection method [30] that is designed for Unmanned Aerial Vehicle (UAV) ultilizes the ORB [31] and descriptor matcher algorithm to align the camera motion caused by the lack of GPS precision or weather variantions. Besides, the CNN-based homography estimation networks are also proposed in both the supervised manner [32] and the unsupervised [15] manner, with more stable image alignment performance when comparing with the traditional SIFT and ORB features based methods. The stable unsupervised homography estimator [15] provides an ideal reference frame alignment method to combine with the DFFnetSeg for pursuing a general foreground segmentation network under the PTZ camera challenge. However, the real-world application of the unsupervised homography estimator [15] focuses more on the UAV and does not consider the foreground influence on the alignment. Therefore, in this paper, a semantic attention mask is proposed to deal with the foreground influence, as the large scale foregrounds appear very frequently in surveillance videos and may cause the wrong prediction of homography matrices. Based on the generality of the foreground segmentation method DFFnetSeg and the unsupervised manner of the homography estimator [15], a deep features homography transformation and fusion network based foreground segmentation method (HTFnetSeg) is proposed to robustly process the unseen videos with PTZ cameras.

## 3. Methodology

Let us denote the *t*-th RGB frame in a *T* frames video by $F^{\left(t\right)}\in R^{h\times w\times 3}$, t∈[1,T], where *h* and *w* are height and width, respectively. The HTFnetSeg aims to produce a foreground mask $M^{\left(t\right)}\in {\left\{0,1\right\}}^{h\times w}$, where 0 denotes the background pixel and 1 denotes the foreground pixel, from the input group which is composed of the current frame $F^{\left(t\right)}$, the previous frames $F^{\left(t-k_{1}\right)}$, and $F^{\left(t-k_{2}\right)}$.

**Figure 1:**
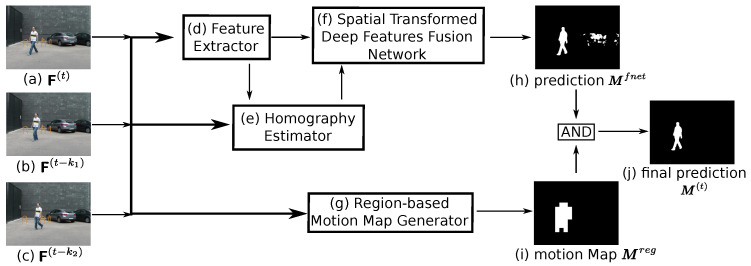
An illustration of the pipeline of HTFnetSeg method. Our method takes a group of frames (**a**–**c**) as input, and produces a binary mask (**j**) as the output. Three major parts (**d**–**f**) construct the HTFnet, which aligns and compares the frames to predict the raw foreground mask (**h**) (up). The post-process step (**g**) further boosts the final prediction to (**j**) by generating the region-based motion map (**i**) (down). The detail functions of (**d**–**f**) are that: (**d**) takes the frames as input and passes the deep features to (**e**,**f**); (**e**) takes the frames and features as input and generates the homography matrices for (**f**); and, (**f**) uses the homography matrices to align the input deep features and finally generates the prediction (**h**).

The HTFnetSeg method consists of four parts, as shown in Figure 1: a feature extractor, a homography extractor, a spatial transformed deep features fusion network (STDFFnet) and a region-based motion map generator.

In detail, the first part is a pre-trained semantic segmentation network PSPNet to extract four deep levels features $F_{l}\left(F^{\left(\cdot \right)}\right)$, l∈[1,4] from the single frame $F^{\left(\cdot \right)}$. The deepest semantic feature maps denoted by $F_{4}\left(F^{\left(\cdot \right)}\right)$ act as the input for semantic attention layer in a homography estimator to generate the semantic attention mask and the rest act as the input for STDFFnet to generate the foreground mask.

The second part is a homography extractor that could generate the homography matrix $H_{12}=H\left(I_{1},I_{2},F_{4}\left(I_{1}\right),F_{4}\left(I_{2}\right)\right)$, which could warp the pixel coordinates of image $I_{1}$ to that of image $I_{2}$. To adapt the unsupervised deep homography estimation model [15] to the PTZ camera foreground segmentation problem, the semantic attention mask is introduced to both the input and the loss function of the original homography estimation model to both reduce the noise that is brought by moving objects and reserve its advantage of unsupervised manner, which can avoid the demand for extra human labelled ground truth. Besides, a mask constraint is proposed for the loss function to avoid the influence of the black boundaries in the warped image.

The third part is a spatial transformed deep features fusion network (STDFFnet), which utilizes the spatial transformation technique [15] to align the input entries of the FusionNet [14] to fulfil its basic assumption that the current frame, previous frame and reference frame share the same scene. The homography matrices extracted between two previous frames and current frame are used to warp the previous frames and the deep features extracted from them to the current frame plane, respectively. Subsequently, the warped frames and features together with the current frame and its features are fed to the FusionNet to generate the predicted foreground mask. Combining the spatial transformation with DFFnet can both tackle the weakness of DFFnet to continuous changing background and benefit from the generality of DFFnet.

The final part is a region-based motion map generator, a post-process step, which is similar to the one in DFFnetSeg method [14] whose function is to eliminate the false positives that are caused by the semantic noise. The semantic noise means that the semantic hint of some regions leads to the misclassification of foreground, because these regions have a high possibility of motion in terms of their semantic classes. The region-level comparison among the input group of frames is also adapted to get the potential motion region, but, different from the corresponding in the DFFnetSeg method, the region-based motion map generator here is based on a conservative strategy. It is because the unavoidable alignment deviation makes the motion map noisy and the conservative strategy is more robust to this noise.

These four parts are discussed in detail as follows.

### 3.1. Feature Extractor

The semantic information acts as an important element for moving object segmentation, as shown in the state-of-the-art moving object segmentation methods [33,34]. In the homography transformation based foreground segmentation methods, the transformed reference video frames are easy to have the deviation when comparing with its corresponding in the current frame plane. Therefore, when the foreground mask is extracted by comparing the reference frames with the current frame, the background regions with relative strong edges are easy to be classified as foreground. Besides, some boundary regions of the current frame cannot find the reference from the previous frames because they may newly appear. In this case, these regions lack the evidence to make the classification decision. Sometimes, even when the reference frames are well aligned without any deviation, the camouflage problem might appear if the foreground object shares a similar colour and intensity with the background region. However, these problems are easy to be tackled if the semantic information are available in the potential moving region. For example, if a “building region” is classified as foreground, then it is easy to be removed based on its semantic feature “building” and, if a “human region” is partly activated, then it is easy to activate all region of this person based on its semantic mask. Therefore, the semantic information extracted by a CNN-based semantic segmentation method PSPNet is used in this paper, but, different from [29], who sets a hard rule to combine the semantic information with foreground mask predictions, the semantic information from the feature extractor is utilized by constructing the semantic attention layer and fusion network.

Specifically, during the homography estimation stage, the correct frame transformation demands that the homography estimator emphasises on the background region because the foreground objects always have different motion type with the camera. The background region can be roughly extracted only based on the semantic information by the semantic attention layer with the deep semantic features as input. Besides, as the FusionNet in STDFFnet demands the usage of the deep features as well, our HTFnetSeg method utilizes a semantic segmentation network, PSPNet, as the feature extractor to extract feature maps for homography estimator and STDFFnet at same time. STDFFnet can be regarded as an extended version of DFFnet for the PTZ camera challenge. Thus, for the STDFFnet part, the feature maps are chosen from the PSPNet in the same way as DFFnet [14], while, for a homography estimator, a deeper layer is chosen to constrain it to only use pure semantic information, as detailed in Section 3.2.

Same as [14], the PSPNet is trained on ADE20K dataset [35,36] without fine-tuning to avoid overfitting. It is because the semantic classes in ADE20K are enough for most surveillance videos and their image domains are also similar. In terms of the architecture, as *w* is 320 and *h* is 240 in HTFnetSeg, the architecture parameters of PSPNet are slightly modified, as shown in Table 1, and its detailed connection is shown in [29]. Batch normalizations are applied after each convolutional layer and the activation function is ReLU.

### 3.2. Homography Estimator

**Figure 2:**
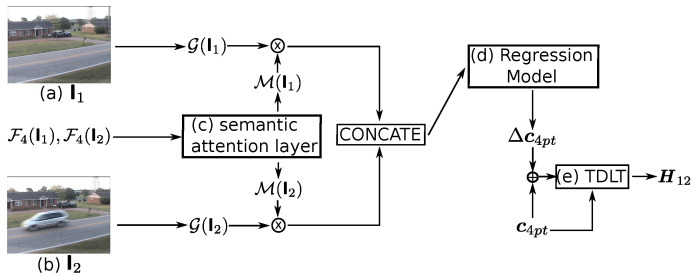
Architecture of the proposed homography estimator. The estimator takes as inputs the paired images (**a**) $I_{1}$ and (**b**) $I_{2}$ and their corresponding deep features $F_{4}\left(I_{1}\right)$ and $F_{4}\left(I_{2}\right)$, and gets as the output the homography matrix $H_{12}$. Specifically, the semantic attention layer (**c**) generates the attention mask $M(I_{1})$ and $M(I_{2})$ based on $F_{4}\left(I_{1}\right)$ and $F_{4}\left(I_{2}\right)$, respectively. Then, the greyscale of images $I_{1}$ and $I_{2}$, denoted by $G(I_{1})$ and $G(I_{2})$, are masked by $M(I_{1})$ and $M(I_{2})$, respectively. After that, the regression model (**d**) takes as inputs the concatenation of the masked greyscale images and predicts as the output the offset of four keypoints, denoted by $\Delta c_{4pt}$. Finally, the TDLT (**e**) obtains the homography matrix based on the 4 keypoints $c_{4pt}$ and its offset $\Delta c_{4pt}$.

To take advantage of the auto feature extraction property of data-driven methods and to avoid the costly ground-truth labelling, the unsupervised deep network is an ideal model for homography estimation. Inspired by a fast and robust homography estimation model [15], a semantic attention based deep homography estimator, denoted by H(·), is proposed to extract the homography matrix, denoted by $H_{12}=H\left(I_{1},I_{2},F_{4}\left(I_{1}\right),F_{4}\left(I_{2}\right)\right)$, when the paired input images are denoted by $I_{1}$ and $I_{2}$, where $F_{4}\left(\cdot \right)$ denotes the output of CONV6 shown in Table 1. The whole architecture of it is proposed in Figure 2. In detail, it consists of three main parts: the semantic attention layer, the regression model, and a differentiable Tensor Direct Linear Transform (TDLT).

The semantic attention layer is a convolutional layer with kernel size 1×1 and two maps followed by a bilinear upsampling layer, which resizes the semantic attention mask to the size of original images. After that, a softmax function is used to distinct the attention region denoted by the class label 1. This layer aims to roughly remove the foreground objects from the input images based on the semantic information. The weights in this layer have the shape of (1,1,150,2), which means that the value of each output neuron is the weighted sum of the 150 semantic predictions from the previous layer. The raw output of the PSPNet is 150 classes, including building, car, water, and so on (details in ADE20k dataset). Obviously, the classes, like “building” and “water”, tend to be regarded as background, which should get higher weights to activate higher values on the neurons denoting the background. However, instead of manually generating a subset of semantically relevant foreground classes, like [34], the semantic attention layer is trained to activate the background region by minimizing the cross-entropy function,

(1) $$L_{m}=-\frac{1}{hw}\sum_{i=1}^{hw}\left[p_{i}\log{\hat{p}}_{i}+\left(1-p_{i}\right)\log\left(1-{\hat{p}}_{i}\right)\right],$$

where $p_{i}$ is the ground truth label and ${\hat{p}}_{i}$ is the output of the prediction at pixel location *i*. After training, the parameters of the semantic attention layer will be frozen for the following training of the regression model. The PSPNet, together with the semantic attention layer, is regarded as an attention mask generator that is denoted by $M\left(\cdot \right)\in R^{h\times w}$.

In terms of the regression model, following the trend of [15], the VGGNet backbone (details in Table 2) is used to regress the offset of keypoints, where the keypoints is the four corners $c_{4pt}\in Z^{8\times 1}$ of the input image. To prepare the input of the regression model, the greyscale of the original image, denoted as G(·), is extracted. Subsequently, given the greyscale image $G(I_{t})$ and the attention mask $M(I_{t})$, the corresponding masked image denoted as Imt is obtained by:

(2) Imt=G(It)⊙M(It),

where t∈{1,2} and ⊙ denote the element-wise multiply. Im1 and Im2 are concatenated as the input of the regression model to obtain the offset $\Delta c_{4pt}$ of the four corners $c_{4pt}$ in $I_{1}$. Therefore, in the plane of $I_{2}$, the corresponding keypoints ${\hat{c}}_{4pt}$ of $c_{4pt}$ is defined as:

(3) $${\hat{c}}_{4pt}=c_{4pt}+\Delta c_{4pt}\;.$$

After regression, a differentiable Tensor Direct Linear Transform (TDLT) layer is applied, as proposed in [15], to solve for the homography matrix $H_{12}\in R^{3\times 3}$ given a set of four 2D points correspondences, $c_{4pt}$ and ${\hat{c}}_{4pt}$, between the plane of $I_{1}$ and $I_{2}$. In this TDLT layer, the last elemant of $H_{12}$ is assumped to be 1. The first 8 elements in $H_{12}$, denoted by ***h***, is calculated by solving the function:

(4) $$Ah={\left[A_{1},A_{2},A_{3},A_{4}\right]}^{T}h={\left[b_{1},b_{2},b_{3},b_{4}\right]}^{T},$$

where given denotation $c_{4pt}={\left[x_{1},x_{2},x_{3},x_{4}\right]}^{T}$, $x_{i}=\left(u_{i},v_{i}\right)$, ${\hat{c}}_{4pt}={\left[{\hat{x}}_{1},{\hat{x}}_{2},{\hat{x}}_{3},{\hat{x}}_{4}\right]}^{T}$, ${\hat{x}}_{i}=\left({\hat{u}}_{i},{\hat{v}}_{i}\right)$, the matrix $A_{i}$ is defined as:

(5) $$A_{i}={\left[\begin{array}{c} 0\;\;0\;\;0\;-u_{i}\;-v_{i}\;-1\;{\hat{v}}_{i}u_{i}\;{\hat{v}}_{i}v_{i}\\ u_{i}\;v_{i}\;1\;\;\;0\;\;\;0\;0\;\;-{\hat{u}}_{i}u_{i}\;\;-{\hat{u}}_{i}v_{i} \end{array}\right]}^{T}\;,$$

and vector $b_{i}$ is defined as:

(6) $$b_{i}=\left[-{\hat{v}}_{i},{\hat{u}}_{i}\right].$$

Based on Equation (4), $H_{12}$ can be solved for using $A^{+}$, the pseudo-inverse of A.

For generally supervised homography estimator, L1 or L2 loss between homography matrix ground truth and its prediction may be chosen as the loss function for the regression model of homography estimator. However, as the homography matrix ground truth is hard to be labelled, even by human and in most circumstances, the homography matrix ground truth is not available in change detection datasets. Therefore, an unsupervised homography estimator seems to be a better choice for the PTZ camera foreground segmentation task. To fulfil the unsupervised training [15], proposed a loss function by comparing the difference between the warped frame with the target frame. Similarly, our loss function is also based on the comparison of the warped frame and target frame, but, to avoid the influence of the foreground objects motion on the offset prediction, a masked loss function that is based on the semantic attention mask is further proposed to only backpropagate the loss belonging to the attention region. That is:

(7) $$L_{h\_m}\left(I_{1},I_{2}\right)=\left\{\begin{array}{cc} \frac{\sum_{i}\left[\hat{M}\left(I_{1}\right)⊙M\left(I_{2}\right)⊙abs\left(\hat{G}\left(I_{1}\right)-G\left(I_{2}\right)\right)\right]}{\sum_{i}\left[\hat{M}\left(I_{1}\right)⊙M\left(I_{2}\right)\right]} & \sum_{i}\left[\hat{M}\left(I_{1}\right)⊙M\left(I_{2}\right)\right]\neq 0\\ 0 & otherwise \end{array}\right.\;,$$

where $\hat{G}\left(I_{1}\right)$ and $\hat{M}\left(I_{1}\right)$ are the warped $G(I_{1})$ and $M(I_{1})$ by the transformation matrix $H_{12}$, and the abs(·) denotes an element-wise absolute value function. Images are warped by a differentiable spatial transformation layer [15] which is based on the two-dimensional (2D) coordinate transformation with bilinear interpolation to enable the training of networks. In addition to focusing on the background region, the loss function can also avoid the influence of the black boundaries of the warped image. In homography estimation networks like [15], they crop the centre of the images as the network input patches and also crop the centre of the warped image to calculate the loss function to avoid the black boundaries in the warped image, but this strategy might cause the wrong prediction in two circumstances: one is when a big foreground object appears in the centre of a surveillance scene and the other is when the black boundaries appear in the cropped region of the warped image because of the large scale scene motion. Benefiting from the masked loss function, our method do not need to crop the images, which enable our method to be robust to those two circumstances, because the foreground objects have a quite lower possibility to cover the most parts of a scene than of the centre region in the scene.

However, when no activated location overlaps between $\hat{M}\left(I_{1}\right)$ and $M(I_{2})$, the masked loss function $L_{h\_m}\left(I_{1},I_{2}\right)$ will be 0, which might lead the model converging to a wrong place. Therefore, a constraint element is proposed as

(8) $$L_{h\_c}\left(I_{1},I_{2}\right)=-\sum_{i}\left[\hat{M}\left(I_{1}\right)⊙M\left(I_{2}\right)\right]\;,$$

for the masked loss function to encourage the larger overlap of activated locations. Subsequently, the overall loss function for homography estimation network is defined as:

(9) $$L_{h}\left(I_{1},I_{2}\right)=L_{h\_m}\left(I_{1},I_{2}\right)+L_{h\_m}\left(I_{2},I_{1}\right)+L_{h\_c}\left(I_{1},I_{2}\right)+L_{h\_c}\left(I_{2},I_{1}\right)+\gamma \left|H_{12}H_{21}-I\right|\;,$$

where as the corresponding for $I_{1}$, $\hat{G}\left(I_{2}\right)$ and $\hat{M}\left(I_{2}\right)$ are the warped $G(I_{2})$ and $M(I_{2})$ by the transform matrix $H_{21}=H\left(I_{2},I_{1},F_{4}\left(I_{2}\right),F_{4}\left(I_{1}\right)\right)$ in $L_{h\_m}\left(I_{2},I_{1}\right)$ and $L_{h\_c}\left(I_{2},I_{1}\right)$. $I\in R^{3\times 3}$ is an identity matrix to restrict the $H_{12}$ to be the inverse of $H_{21}$, which could enhance the stability of the homography prediction, where *γ* is used to control the strength of this restrict (γ=0.01 in our experiment). The parameters of the regression model in our semantic attention based homography estimator can be optimized by minimizing $L_{h}$.

### 3.3. Spatial Transformed Deep Features Fusion Network

**Figure 3:**
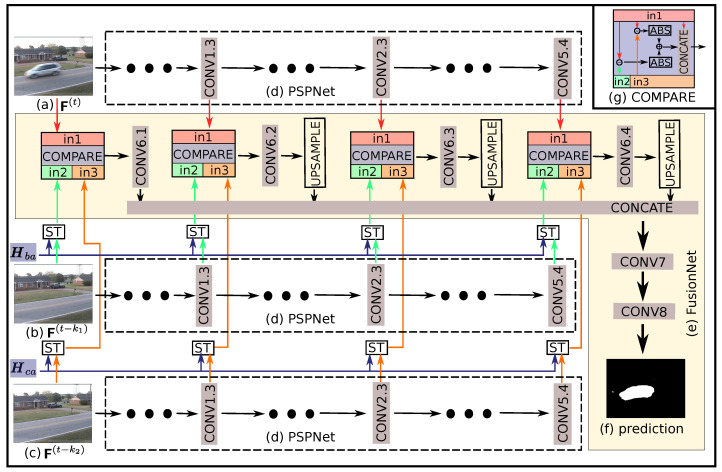
The architecture of our proposed STDFFnet. Firstly, features from chosen layers of PSPNet (**d**) are extracted, given inputs, including a current frame (**a**), previous frames (**b**,**c**), respectively. After that, the spatial transformations (ST) that are based on matrices $H_{ba}$ and $H_{ca}$ are conducted for the features from corresponding chosen layers. The spatial transformed feature maps, instead of the raw feature maps as used in DFFnet, are used as the inputs of the FusionNet (**e**). Finally, based on the features from (**a**) and the transformed features from (**b**,**c**), the FusionNet generates the foreground mask (**f**). The architecture of the comparing operator (COMPARE) is shown in the upper-right corner.

The deep feature fusion network (DFFnet) [14] has shown its robustness and generality for foreground segmentation tasks, but its performance relies on the assumption that the reference frame, previous frame and current frame are from the same scene. The DFFnet can tackle the sudden scene change by reference frame update strategy, but it has difficulty to deal with the continuous scene change situation. A spatial transformed deep features fusion network (STDFFnet) is proposed in order to both fully exploit the advantage of DFFnet and overcome its weakness on continuous scene change. Its inputs consist of the current frame $F^{\left(t\right)}$, the previous frames $F^{\left(t-k_{1}\right)}$, and $F^{\left(t-k_{2}\right)}$, and the homography matrices $H_{ba}=H\left(F^{\left(t-k_{1}\right)},F^{\left(t\right)},F_{4}\left(F^{\left(t-k_{1}\right)}\right),F_{4}\left(F^{\left(t\right)}\right)\right)$ and $H_{ca}=H\left(F^{\left(t-k_{2}\right)},F^{\left(t\right)},F_{4}\left(F^{\left(t-k_{2}\right)}\right),F_{4}\left(F^{\left(t\right)}\right)\right)$. Its architecture is shown in Figure 3. Specifically, the deep features are firstly extracted by PSPNet, being denoted by $F_{l}\left(F^{\left(\cdot \right)}\right)$, l∈[1,3] corresponding to the output of CONV1.3, CONV2.3, and CONV5.4 layers of the PSPNet, respectively. The $H_{ba}$ and $H_{ca}$ are the estimation of the homography matrices, which can map the pixel coordinates of $F^{\left(t-k_{1}\right)}$ and $F^{\left(t-k_{2}\right)}$ plane to the corresponding of $F^{\left(t\right)}$ plane. As the deep convolutional neural network reserves the spatial distribution of the input image, and the coordinates are normalized to range [−1,1] [15] in the spatial transformation layer, the homography matrices $H_{ba}$ and $H_{ca}$ can be used to map the 2D coordinates of each single feature map. Therefore, feature maps are directly transformed rather than being re-extracted from the warped image, because this strategy can reuse the features extracted during the attention mask generation step to reduce the computational complexity of HTFnetSeg. As the original frames are also regarded as a kind of features, here, for the unity of denotation, the original frames $F^{\left(\cdot \right)}$ are denoted by $F_{0}\left(F^{\left(\cdot \right)}\right)$. Subsequently, the transformed features ${\hat{F}}_{l}\left(F^{\left(t-k_{1}\right)}\right)$ and ${\hat{F}}_{l}\left(F^{\left(t-k_{2}\right)}\right)$, together with features of the current frame $F_{l}\left(F^{\left(t\right)}\right)$, where l∈[0,3], act as the input set of the FusionNet, getting the foreground mask prediction $M^{fnet}\in {\left\{0,1\right\}}^{h\times w}$ (where 1 denotes foreground) for frame $F^{\left(t\right)}$. In STDFFnet, only the FusionNet part has parameters to be trained by the change detection data. The detail hyper-parameters of FusionNet is shown in Table 3, and, in FusionNet, the batch normalization layer is used after each convolutional layer and the ReLU activation function is used (see [14] for more implementation details of the FusionNet part).

The cross-entropy loss that is described in Equation (1) is a widely used loss function to optimize the classification model, whereas, in HTFnet, the regression model and the fusion network are aimed to be trained in an end-to-end manner, because, as a homography transformation based foreground segmentation network, the HTFnet demands the homography estimator to produce an applicable matrix ***H*** for foreground segmentation task. Thus, the union loss function is defined as:

(10) $$L=\alpha L_{m}+L_{h}\left(F^{\left(t-k_{1}\right)},F^{\left(t\right)}\right)+L_{h}\left(F^{\left(t-k_{2}\right)},F^{\left(t\right)}\right)\;,$$

where $L_{m}$ is the cross-entropy loss for training the mask $M^{fnet}$ and *α* is used to balance the backpropagation from the loss function for mask prediction and homography matrix prediction. In our experiment, *α* is 100, which enables the $L_{m}$ to have the same order of magnitude as $L_{h}$ and to be slightly higher than $L_{h}$ to pay more attention to optimizing the mask prediction loss, because, during the experiment, the homography estimator is first pre-trained by Equation (9) before end-to-end training and only need to be fine-tuned during this stage.

### 3.4. Region-Based Motion Map

The fusion network is easy to get the false-positive classification, which suffers from the semantic noise, as mentioned in DFFnetSeg [14]. Therefore, a region-based motion map is proposed to reduce the influence of the semantic noise. However, in the PTZ camera video, the scene which may keep changing is more dynamic than the situation the DFFnetSeg designed for. The motion map generated by the greedy strategy proposed in [14] may activate a large amount of static regions, which is caused by the deviation of the frame alignment. Although, as shown in [15], the unsupervised homography estimation method can outperform the traditional methods, such as Scale Invariant Feature Transform (SIFT) and enhanced correlation coefficient (ECC), the deviation might still exist between the current frame and the transformed frames, especially near the edges. A conservative strategy is proposed to extract a region-based motion map from the input group consisting of the current frame $F^{\left(t\right)}$ and the warped previous frames ${\hat{F}}^{\left(t-k_{1}\right)}$ and ${\hat{F}}^{\left(t-k_{2}\right)}$ to reduce the effect of this deviation on the region-based motion map.

In detail, given denotation $F^{\left(t\right)}$, ${\hat{F}}^{\left(t-k_{1}\right)}$ and ${\hat{F}}^{\left(t-k_{2}\right)}$, the pixel level difference maps D1 and D2 are defined by:

(11) $$\begin{array}{c} D1_{i,j}=\left\{\begin{array}{ll} 1 & if\;\exists k,\;|F_{i,j,k}^{\left(t\right)}-{\hat{F}}_{i,j,k}^{\left(t-k_{1}\right)}|>\theta ,\;where\;\left(i,j\right)\notin B_{1}\\ 0 & otherwise \end{array}\right.,\\ D2_{i,j}=\left\{\begin{array}{ll} 1 & if\;\exists k,\;|F_{i,j,k}^{\left(t\right)}-{\hat{F}}_{i,j,k}^{\left(t-k_{2}\right)}|>\theta ,\;where\;\left(i,j\right)\notin B_{2}\\ 0 & otherwise \end{array}\right., \end{array}$$

where (i,j) denotes the location of one pixel. *k* denotes the colour channel. $B_{1}$ and $B_{2}$ are the sets of 2D coordinates of the black boundary regions in the warped frames ${\hat{F}}^{\left(t-k_{1}\right)}$ and ${\hat{F}}^{\left(t-k_{2}\right)}$, respectively. The black boundaries are caused by the situation that the corresponding 2D coordinates in the warped image are outside the original image boundaries. A location is activated when a recognized difference exists in any colour channel, which can be more robust to camouflage problem than only considering the grey intensity. To reduce the noise that is caused by the edge deviation, the erosion and dilation techniques are used to post-process the D1 and D2, with a 5×5 disk-shaped kernel. To further reduce the influence of deviation of the alignment, the pixel level motion map $M^{pix}$ is generated in a relatively conservative strategy, which is defined as:

(12) $$M_{i,j}^{pix}=\left\{\begin{array}{ll} 1 & if\;D1_{i,j}=1\;and\;D2_{i,j}=1\\ 0 & otherwise \end{array}\right..$$

Thus, one location is only activated when a strong hint of motion is indicated by the difference maps. However, this strategy might result in the holes in the motion region, which are caused by the foreground overlapping. The region block can well fill in these holes depending on activation situation of the neighbours as in [14]. The implementation details is that the whole motion map is divided into regions with size N×N without overlapping (the boundaries are padded to be valid), denoted by $M_{k}\subseteq M^{pix}$ (with ${⋃}_{k}M_{k}=M^{pix}$). Subsequently, the region-based map $M^{reg}$ is obtained according to the quantity of motion in each region, as follows:

(13) $$\begin{array}{c} M_{\Psi_{k}}^{reg}=\left\{\begin{array}{ll} 1 & \mathrm{if}\;\sum_{\left(i,j\right)\in \Psi_{k}}M_{i,j}^{pix}>\beta \\ 0 & \mathrm{otherwise} \end{array}\right.,\\ \mathrm{with}\;\Psi_{k}=\left\{\left(i,j\right)|M_{i,j}^{pix}\in M_{k}\right\}. \end{array}$$

One region block of the map $M^{reg}$ is activated when the quantity of motion in that specific region is larger than *β*.

Given the region-based motion map $M^{reg}$, the final foreground prediction $M^{\left(t\right)}$ is defined by:

(14) $$M_{i,j}^{\left(t\right)}=\left\{\begin{array}{ll} 1 & \mathrm{if}\;M_{i,j}^{reg}=1\;\mathrm{and}\;M_{i,j}^{fnet}=1\\ 0 & \mathrm{otherwise} \end{array}\right..$$

**Figure 4:**
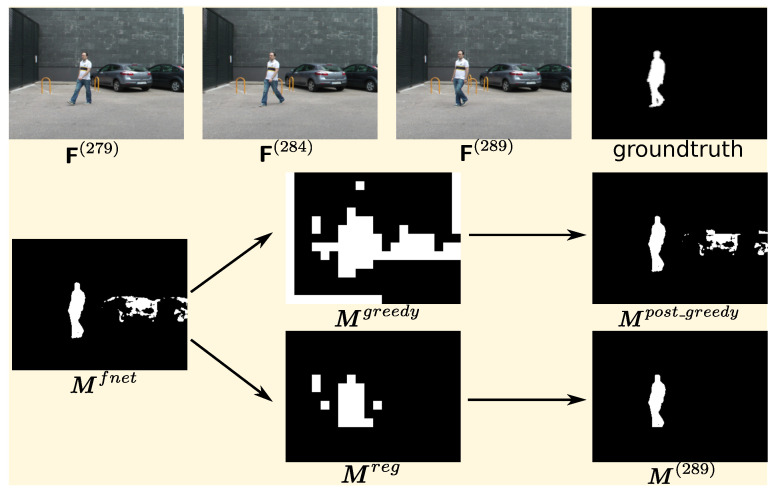
A sample of the performance of post-process. In this sample, parameters $\left(t,k_{1},k_{2},\theta ,\beta ,N\right)=\left(289,5,10,20,10,64\right)$, respectively. $M^{fnet}$ is the predicted foreground mask for the frame $F^{\left(289\right)}$, which is also the raw output of the fusion network with the input three frames shown in the first raw. $M^{greedy}$ is the region-based map proposed in a greedy strategy from the DFFnetSeg method, and $M^{reg}$ is the conservative one proposed in this paper. $M^{post\_greedy}$ and $M^{\left(289\right)}$ are post-processed final predictions by these two maps, respectively.

Figure 4 shows a sample of the post processing effect. As shown in the figure, in the raw output of the fusion network, the regions with the cars are quite noisy, because cars have a high possibility to be foreground in terms of semantic information, but, in the current frame, the cars are parked there. Therefore, the region-based motion map is used to enhance the motion hint for the final decision. As the greedy motion map generation strategy proposed in [14] is designed for relatively static scenes, the greedy motion maps are not clean enough to eliminate the false positive regions of fusion network prediction for PTZ camera videos. The conservative motion map is still not perfectly clean, with some false positives being caused by alignment deviation, but it seems good enough to remove the false positive regions in the output of the FusionNet, as the FusionNet is robust to the reference frame alignment deviation in the regions who semantically have the low possibility to be foreground, as shown in the Figure 4. Therefore, the combination of the region-based motion map and FusionNet can overcome the drawback of each other.

## 4. Experimental Setting

### 4.1. Dataset

The performance of our HTFnetSeg method is evaluated by PTZ camera related video sequences in two dataset CDnet2014 [26] and LASIESTA [37].

The CDnet2014 dataset is the largest change detection benchmark, including 53 video sequences categorized to 11 challenges. However, most of the videos are captured by stationary cameras. As the HTFnetSeg method aims to deal with the continue or intermittent panning, tilting, zooming camera situation, the videos “continuousPan”, “intermittentPan”, and “zoomInZoomOut” are chosen from the PTZ camera category of CDnet2014 to test our method. Additionally, the selected videos from other categories are used to train the homography estimator and the fusion network of the HTFnetSeg method. The selection of videos is same as [14]. For each video sequence, CDnet2014 provides the pixel-level ground truth for a subset of it, so only the labelled frames are included in the training and testing set in this experiment.

Besides, seven objective evaluation metrics are provided by CDnet2014 to evaluate the performance of algorithms quantitatively. Here, the three most popular metrics are chosen for this experiment:

- Recall (R): TP / (TP + FN).
- Precision (P): TP / (TP + FP).
- F-Measure (F): (2 * Precision * Recall) / (Precision + Recall).

The LASIESTA dataset provides 17 real indoor and 22 outdoor sequences organized in 12 categories, where all of the videos related to PTZ camera moving are chosen as the testing set of this experiment and no other sequence is chosen for training, because the sequences chosen from CDnet2014 are various enough to train a general model. In detail, the chosen videos are “I_MC_01”, “I_SM_01”, “I_SM_02”, “I_SM_03”, “O_MC_01”, “O_SM_01”, “O_SM_02”, and “O_SM_03” belonging to moving camera (MC) and simulated motion (SM) categories, respectively. “I_SM_01”, “I_SM_02” and “I_SM_03” are videos recorded in an indoor scene with the low, medium, and high strength of camera panning, respectively. “O_SM_01”, “O_SM_02”, and “O_SM_03” are videos recorded in an outdoor scene with the low, medium, and high strength of camera panning and tilting, respectively. As the pixel-level ground truth is available for all of the frames in LASIESTA, the whole sequences for chosen videos are used.

### 4.2. Training and Testing Set

Many state-of-the-art CNN-based change detection algorithms choose 50% to 80% or fixed number (i.e., 50, 100) frames from each single video sequence as the training set, with the rest frames from these sequences as the testing set. This separation cannot test the generality of methods for unseen scenes and objects, because the same background scenes and foreground objects may coexist in both the training and testing set. To tackle this drawback, in this experiment, the training set and the testing set are generated in a way that enables that no same background scenes and foreground objects coexist in both the training and testing sets.

Only the chosen videos in CDnet2014 are used as the training set, as mentioned in Section 4.1. The training videos chosen from CDnet2014 are same as the experiment in [14]. However, they are all captured by the stationary cameras, so, to train the neural network for PTZ cameras, they are augmented by the homography transformation technique to simulate the panning, tilting, and zooming camera videos, respectively. In each augmented video, 50 groups of frames with the interval $(k_{1},k_{2})\in \left\{(5,10),(10,20),(20,40),(40,80)\right\}$ are randomly chosen to enable the trained model to adapt to the various scales of camera motion, as under most camera moving circumstances, the larger the interval is, the smaller the overlap of two scenes is.

For testing, 13 videos are chosen from two datasets and they are classified into three categories based on the motion pattern of cameras, including panning (Pan), panning and tilting (P&T), and zooming in and zooming out (Zoom). The categories and chosen frame indices details are shown in Table 4. The $\left(k_{1},k_{2}\right)$ is set to (5,10) during testing.

### 4.3. Compared Methods

To evaluate the accuracy of HTFnetSeg method, is compared with the following state-of-the-art algorithms:

- PAWCS [5] and SWCD [18], the high-rank traditional background subtraction methods on CDnet2014 in terms of the PTZ camera category with source code opened to the public,
- SCBU [23], a traditional moving object detection method designed for a moving camera whose binary executable file is available online,
- MLBS [24], a background subtraction method for freely moving cameras based on motion trajectories clustering with source code opened to the public
- FgSegNet [12], the top CNN-based foreground segmentation method on CDnet2014 with source code opened to the public, and
- ICNET-CDNET [28], the deep change detection algorithm based on the collaboration of an image completion network and a change detection network,

on the dataset mentioned above. To ensure a fair comparison between the supervised models, all of the supervised models in this experiment are trained on the same data, the training set described in Section 4.2. Besides, the hyper-parameters of both supervised methods and traditional methods are the same as the ones that are described in the source code and papers. The pre-trained model is also implemented to initialize the model parameters according to the original paper.

### 4.4. Implementation Details

This experiment is implemented based on TensorFlow framework on a single NVIDIA GeForce GTX 1080ti GPU. In this experiment, the HTFnet is trained end-to-end by Equation (10) based on the labelled change detection data, after a series of pre-training. Specifically, the feature extractor PSPNet is pre-trained on ADE20K as in [29] and no parameter inside the PSPNet is fine-tuned after that. As the regression model in homography estimator depends on a good semantic attention mask generator, the semantic attention layer is trained with 24 epochs by the labelled change detection data in the training set (where the background regions in ground truth masks are set to be class 1 and others are 0), which enables the layer to learn to decide whether to pay attention to a region or not only based on the semantic information of a single frame. After that, the parameters of the semantic attention layer are frozen during the training of the HTFnet, because their job is to produce an attention mask that can tolerate the wrong classification to roughly remove objects who have the possibility to move. The reference frame features of the FusionNet are supposed to be the spatial transformed features matching to the current frame plane, which demands that the proper homography matrices are available. The regression model in homography estimator is first trained by minimizing the corresponding loss $L_{h}$ with 54 epochs in order to enable the parameters of the fusion network to converge stably. Subsequently, the whole HTFnet is trained by minimizing the loss function L with six epochs, which optimizes the fusion network and fine-tunes the homography estimation at the same time. As the preprocessing, the size of frames are standardized to 240×320 and the frames are normalized by subtracting by the mean. It is worth noting that the stride of CONV1.1 in the pre-trained PSPNet is changed, but it still does not need fine-tuning because the CNN-based model is robust to the scale change. During training, the initial learning rate is $5\times 10^{-5}$ and the RMSProp is used for optimization.

In order to assess the HTFnetSeg for the parameters *θ*, *N* and *β* in region-based motion map stage mentioned in Section 3.4, the foreground masks generated from FusionNet are post-processed by the motion maps using each combination of θ∈{20,50,80,110}, N∈{8,16,32,64}, and β∈{5,10,20,40}. These three parameters of HTFnetSeg method provide enough flexibility to boost the foreground mask for various motion pattern in video sequences. The raw output of the fusion network mentioned in Section 3.3 is with average F-Measure 0.7349. When the motion map generator that is mentioned in [14] is used, the best result is 0.7796 with (θ,N,β)=(50,32,40). However, when the conservative strategy mentioned in Section 3.4 is used, the region-based motion map achieves a better boost effect whose corresponding F-Measure is 0.8135, with (θ,N,β)=(20,64,10). From the corresponding parameters for the best F-Measure, it is noticed that the conservative strategy demands the relatively loose parameters and vice versa. For example, in our conservative strategy, a pixel is regarded as moving when the difference is bigger than 20, whereas it would be better to be more than 50 when using the motion map generator in [14], which shows the trade-off between the strictness of the strategy and parameter values. The results show that the conservative strategy is more applicable to the PTZ camera situation under this HTFnetSeg architecture and the same conclusion can also be obtained from Figure 4. In detail, when the three parameters are in the range shown in Table 5, the region-based motion map can improve the final foreground mask to a different degree. The table shows that θ=110 is too large to detect the motion and θ=20 seems to be more guaranteed, because, when θ=20, almost all of the combinations of the *N* and *β* boost the final results, except for (8,40).

## 5. Results

### 5.1. Comparison

The performance is compared among methods on the testing data quantitatively and qualitatively. The parameter setting is as described in Section 4, where (θ,N,β)=(20,64,10).

From the perspective of quantitative evaluation, precision, recall, and F-Measure are used. According to the definition of these metrics, the precision pays greater attention to indicate the false positives, the recall pays more attention to indicate the false negatives, and the F-Measure considers both of these two factors. The “Mean” values in Table 6 denote the average values over all of the test videos. Our method almost achieves the best performance in all categories and evaluation metrics, except for the precision of P&T category, as shown in Table 6. In detail, the F-Measure values of HTFnetSeg are 40.59%, 4.43%, and 56.53% greater than the second best results in Pan, P&T, and Zoom categories, respectively. The overall F-Measure is also 30.67% higher than the second one. The SCBU and MLBS methods obtain the higher precision than HTFnetSeg in the P&T category, but their F-Measure is lower than ours because their recall is quite low, which means that these methods suffer greatly from the false-negative classification. In terms of traditional methods, the background update based method SCBU and the motion trajectories based MLBS outperform other traditional methods. Additionally, in terms of the supervised deep learning method, the single image based FgSegNet performs better than the ICNET-CDNET. The free-moving camera change detection method ICNET-CDNET seems to perform abnormally on the testing videos, with quite low metrics values. It is not because the ICNET-CDNET is not suitable for these videos but because this experiment aims to test the performance of methods on unseen videos and the ICNET has difficulty in reconstructing the background for one video if no background frame in that video is used for training. Therefore, the poor performance of ICNET-CDNET here shows its weakness in generality. The evaluation metrics for the Zoom category of MLBS method are not available, because the experiment shows that the MLBS method only produces negative results for all of the pixel locations in the frames from the “zoomInZoomOut” video, which causes both TP and FP are 0. This might be because the motion pattern of zooming is hard to be clustered based on the difference between trajectories in motion and spatial location. Therefore, although MLBS achieves the competitive result when comparing with other methods, it is still not general enough for different motion patterns.

**Figure 5:**
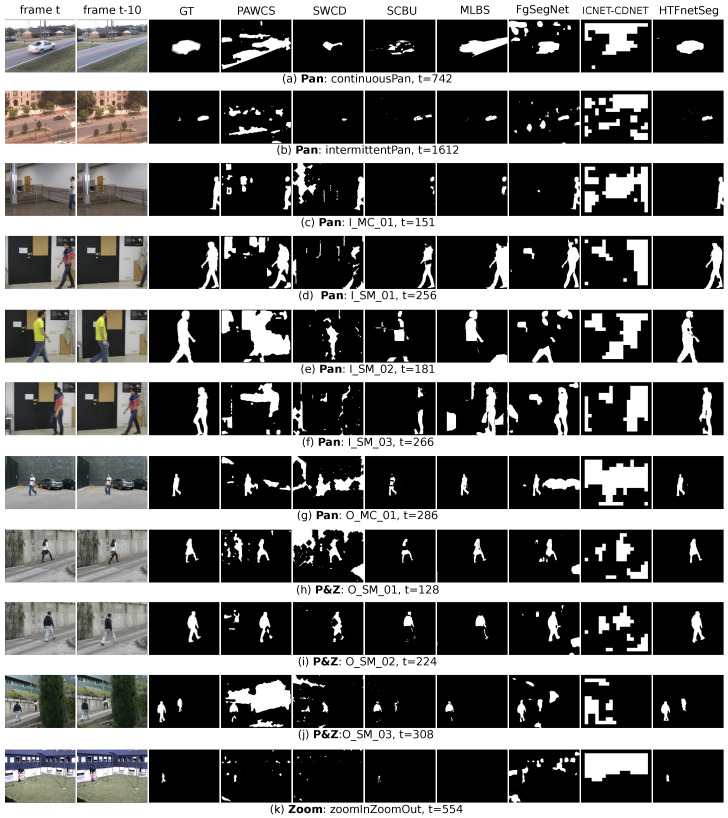
Qualitative results comparison on the seven algorithms. The first column is the current frame $F^{\left(t\right)}$ and the second column is one of the reference frames $F^{\left(t-10\right)}$.

The qualitative details are shown in the sampled visualization Figure 5, where one frame from each sequence is chosen. The HTFnetSeg almost outperforms others in all samples, except getting the similar results with the MLBS in Figure 5b,g,h, as shown in the figure. The result in Figure 5f shows that, when the background and the foreground share a quite similar colour, our difference based method is still affected by the camouflage problem (there is a misclassification region on the shank), but, as shown in Figure 5j, the robustness of our method to the camouflage problem is higher than most of the methods. In contrast, FgSegNet is not influenced by the camouflage problem, because it generates the foreground mask only based on a single frame other than the comparison. Therefore, it detects the foreground object highly depending on the appearance of the object, which might cause the false positives, such as the parked cars in Figure 5g, which is extracted because cars have a high possibility to move when only considering one frame. Except for HTFnetSeg, the SCBU seems to obtain the best performance from the perspective of visualization, as it almost catches all of the moving objects with relatively less false positives and the main weakness of it is those missing foreground parts. MLBS shows its main drawback of instability, which shows better performance than SCBU in some sequences, but, in sequences like Figure 5a,f, it shows more false positives and, even in Figure 5k, it can not catch the foreground at all. The results of PAWCS here are not as good as the ones that are provided by the CDnet2014, because the results on the CDnet2014 used the additional frames for method initialization. The PAWCS might perform better if the background pixel value of the current frame is available in its background model, but, during this comparison experiment, to play fair, no additional data are used from the test videos to boost the performance. As the PAWCS and SWCD are not designed for moving camera and they are just more robust to the moving camera, when comparing with other static camera methods, the performance of them is worse than the other traditional change detection methods designed for moving cameras. Noticeably, the ICNET-CDNET performs even worse than PAWCS. As announced above, they need the background frames from the test scene to train the ICNET to generate a good reference frame, but the background frames are not available in this experiment, because the experiment aims to evaluate the generality of methods to unseen videos. Additionally, the prediction of ICNET-CDNET is block-based, which can deal with some noise at the cost of losing precision.

In conclusion, the HTFnetSeg method outperforms state-of-the-art methods by a great gap in both quantitative evaluation and qualitative evaluation.

### 5.2. Ablation Studies

In this subsection, a series of ablation tests are conducted by comparing the performance of the DFFnetSeg algorithm with our algorithm and also by testing the effect of adding motion map mask and fine-tuning homography estimator, respectively. It is worth noting that here that the homography estimator fine-tuning is used to further discover the ability of our HTFnetSeg and it is not used in the experiment described above. As shown in Table 7, our raw HTFnet can already outperform the DFFnetSeg by 14.97% in F-Measure, which shows that the proposed elements that are designed for the PTZ camera challenge can extend the ability of DFFnet to this challenge effectively. Additionally, the DFFnetSeg shows higher recall and lower precision than our algorithm, which illustrates that it easy to produce false-positive classification that is caused by the background change. In terms of the inner design of the HTFnetSeg, the F-Measure is improved by 7.86% when the motion map mask is conducted on original prediction of HTFnet with parameters (θ,N,β)=(20,64,10) and the improvement comes from the increase of precision by 12.42%, which corroborates that the motion map mask can greatly reduce the false-positive classification. Besides, the performance of HTFnet is attempted to be improved further by fine-tuning the homography estimator. Based on the characters of the different parts of the homography estimator, only the unsupervised regression model is fine-tuned in this step. Therefore, only 10 groups of frames are randomly chosen from each testing sequence with the parameters $\left(k_{1},k_{2}\right)=\left(5,10\right)$ to fine-tune the regression model in homography estimator. Even the frames in the testing set are used during the fine-tuning stage, but still, no human labelled data from the testing set are used. The main effect of fine-tuning is the increase of the precision by 5.98%, which benefits from the decrease of the false positives caused by the deviation of image alignment. After being further processed by the region-based motion map, the precision is also improved by 12.1%, which is similar to the effect of the motion map mask on the original prediction, 12.42%. It indicates the amount of semantic noise influence that could be eliminated by the motion map. When both fine-tuning and motion map steps are conducted, the precision increases 18.08% which boosts the F-Measure by 11.52%, with only recall reducing by 3.83%. The reason for the recall decrease is that, in some motion regions, the motion hint is too weak to catch by the motion map. Overall, the fine-tuning and motion map are both effective in boosting the final prediction and easy to conduct (no additional human label needed), which is worth being applied in the real-world situation.

## 6. Discussion

The primary purpose of this paper is to describe a novel deep learning based foreground segmentation method for the surveillance videos recorded by the PTZ cameras. As a deep learning based method, our HTFnetSeg demonstrates its advantage of high-quality performance when comparing with the state-of-the-art traditional methods, as shown in Table 6. The table also shows the better generality of the HTFnetSeg comparing with other deep learning based methods because the HTFnetSeg outperforms them when the test scenes and objects are unseen during the training stage. This advantage widens the usage field of the HTFnetSeg, because this testing condition is common in real-world usage. The design of the HTFnetSeg is motivated by overcoming the drawback of DFFnetSeg [14] on the PTZ camera challenge. Therefore, an unsupervised homography estimator is combined to align the reference frames without extra training labels. The comparison in Table 7 shows that the HTFnetSeg performs much better than DFFnetSeg under the PTZ camera challenge. These facts indicate that our method architecture and training strategy work effectively as they supposed to be.

The mechanism of our HTFnetSeg distinguishing the foreground from the background region is comparing the current frame with the reference frames. Different from other comparison-based methods [5,28], who assume that the reference frames are pure background with as few foregrounds as possible, our method tolerates the existent of foreground objects on the reference frames, but is under the assumption that the foreground objects moved during the current frame and the reference frames. In future work, the image completion network from [28] might be considered to improve the reference frame to further deal with the restrain brought by this assumption. It based on the following reason. As analysed in Section 5.1, ICNET in [28] does not work well on unseen videos, because no background frame in that video is used for training. However, if the background patches classified by our HTFnetSeg is used as the training set of ICNET, it is possible to enable it to reconstruct a good background reference.

**Figure 6:**
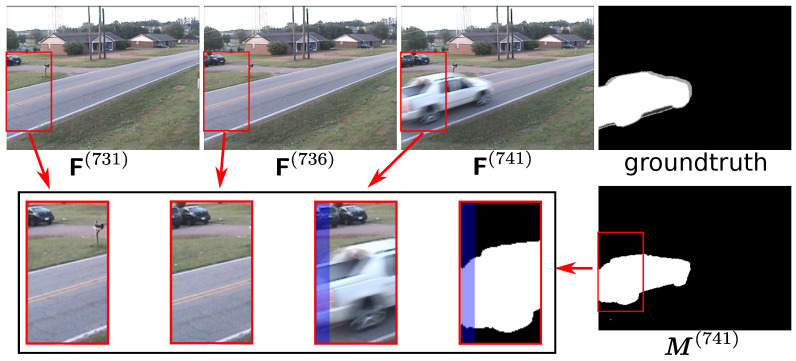
A sample when there is no matched region from the reference frames for the boundary of the current frame. In detail, $M^{\left(741\right)}$ denotes the predicted final foreground mask for the current frame $F^{\left(741\right)}$, with the reference previous frames $F^{\left(736\right)}$ and $F^{\left(731\right)}$. The images in the bottom left corner are the enlarged version of the details in red boxes, respectively. The region covered by blue highlights the region without a match.

An additional point to be discussed is that the black boundary would appear in the warped reference frames because of the background scene motion. In the comparison-based change detection method, the black boundary might cause a lack of evidence to make a decision in these specific regions. In this case, our method takes advantage of semantic information and spatial connection in the deep network, which can predict the foreground mask, even when some reference region is not available. For example, as shown in Figure 6, the region that is covered by blue illustrates that in our method when some part of the boundary region can not find the matched region in reference frames, the foreground mask in that region can still be predicted to some extent.

As can be observed in Table 6 and Figure 5, MLBS [24] is designed for freely moving camera and achieves the second-best mean F-Measure, but it fails to extract the foreground for the “zoomInZoomOut” video. It might be because MLBS is based on the motion trajectory clustering and for panning and tilting, the motion trajectories of background tend to be similar, but, for zooming, the background pixels may move apart from or toward a focus centre which causes the trajectories of background to be different and hard to be clustered. In contrast, the compensation-based method SCBU [23] is more robust on different camera motion pattern categories, because its projective transformation matrix could compensate the camera motion effectively. Therefore, for the PTZ camera challenge, the compensation-based methods seem to be more robust to its camera motion pattern. Similarly, our HTFnetSeg also benefits from compensating the camera motion by image alignment.

## 7. Conclusions

We propose an end-to-end foreground segmentation neural network HTFnetSeg for PTZ cameras, where the homography estimation network is combined with the deep feature fusion network to extend the application of the DFFnetSeg to a wider camera motion situation by conquering its weakness on the continuous moving camera situation. The semantic attention based homography estimator is proposed to reduce the influence of the foreground motion and provide the more stable homography matrices for reference frames alignment. The spatial transformed deep feature fusion network acts as the extended version of DFFnetSeg to align the reference features and produce the foreground mask universally based on the comparison. The usage of the deep features from the pre-trained semantic segmentation model maintains the generality of our network and the feature reusing strategy also reduces the computational load. Finally, as the post-processing part the conservative strategy for region-based motion map further reduces the false positives that are caused by the semantic noise.

In order to make an accurate evaluation, the proposed method is compared with the state-of-the-art approaches on multiple challenging videos provided by two public datasets CDnet2014 and LASIESTA. Quantitative and qualitative experiments illustrate that the proposed method achieves promising performances in term of these two aspects, respectively. Besides, ablation studies are also conducted to evaluate the effect of the components designed for the proposed method. The results demonstrate that all of these components promote our method from different aspects to work robustly and universally to the unseen videos with PTZ cameras.

The homography matrix estimated in HTFnetSeg is designed for the planar spatial transformation, which seems to be enough for common PTZ surveillance videos whose camera motion could be modelled by a planar transformation. In future, our homography estimator has the potential to be extended to a multi-layer homography model to adapt to other moving camera situation where the camera may be displaced. Subsequently, the foreground segmentation framework of our HTFnetSeg could be further extended to the free-moving camera situation.

## Figures and Tables

**Table 1:** The details of the architecture of the modified PSPNet.

ResNet			Pyramid Pool Module		
layer name	output size	layers info	layer name	output size	layers info
CONV1.1	240×320	3×3,64,stride1	pool1	1×1	60×80avgpool,stride60×80
CONV1.2	240×320	3×3,64,stride1	pool1_conv	1×1	1×1,512,stride1
CONV1.3	240×320	3×3,128,stride1	upsample1	60×80	bilinear
CONV2.x	120×160	3×3maxpool,stride2	pool2	2×2	30×40avgpool,stride30×40
		$\left\{\begin{array}{c} 1\times 1,\;64\\ 3\times 3,\;64\\ 1\times 1,\;256 \end{array}\right\}\times 3$	$\begin{array}{c} pool2\_conv\\ upsample2 \end{array}$	$\begin{array}{c} 2\times 2\\ 60\times 80 \end{array}$	$\begin{array}{c} 1\times 1,512,stride\;1\\ bilinear \end{array}$
CONV3.x	60×80	$\left\{\begin{array}{c} 1\times 1,\;128\\ 3\times 3,\;128\\ 1\times 1,\;512 \end{array}\right\}\times 4$	$\begin{array}{c} pool3\\ pool3\_conv\\ upsample3 \end{array}$	$\begin{array}{c} 4\times 4\\ 4\times 4\\ 60\times 80 \end{array}$	$\begin{array}{c} 15\times 20\;avgpool,\;stride\;15\times 20\\ 1\times 1,\;512,stride\;1\\ bilinear \end{array}$
CONV4.x	60×80	$\left\{\begin{array}{c} 1\times 1,\;256\\ 3\times 3,\;256\\ 1\times 1,\;1024 \end{array}\right\}\times 6$	$\begin{array}{c} pool4\\ pool4\_conv\\ upsample4 \end{array}$	$\begin{array}{c} 10\times 10\\ 10\times 10\\ 60\times 80 \end{array}$	$\begin{array}{c} 6\times 8\;avgpool,\;stride\;6\times 8\\ 1\times 1,\;512,stride\;1\\ bilinear \end{array}$
CONV5.x	60×80	$\left\{\begin{array}{c} 1\times 1,\;512\\ 3\times 3,\;512\\ 1\times 1,\;2048 \end{array}\right\}\times 3$	$\begin{array}{c} CONV5.3\\ CONV5.4\\ CONV6 \end{array}$	$\begin{array}{c} 60\times 80\\ 60\times 80\\ 60\times 80 \end{array}$	$\begin{array}{c} concatenation\\ 3\times 3,\;512,stride\;1\\ 1\times 1,\;150,stride\;1 \end{array}$

**Table 2:** The details of the architecture of the regression model.

Layer Name	Output Size	Layers Info	Layer Name	Output Size	Layers Info
conv1.1	240×320	3×3,64,stride2	pool3	15×20	2×2maxpool,stride2
conv1.2	120×160	3×3,64,stride1	conv4.1	15×20	3×3,128,stride1
pool1	60×80	2×2maxpool,stride2	conv4.2	15×20	3×3,128,stride1
conv2.1	60×80	3×3,64,stride1	dropout	15×20	0.5
conv2.2	60×80	3×3,64,stride1	flattern	38,400	−
pool2	30×40	2×2maxpool,stride2	fc1	1024	−
conv3.1	30×40	3×3,128,stride1	fc2	8	−
conv3.2	30×40	3×3,128,stride1			

**Table 3:** The details of the hyper-parameter setting of the FusionNet.

Layer Name	Output Size	Layers Info	Layer Name	Output Size	Layers Info
conv6.1	240×320	3×3,32,stride1	upsample	240×320	bilinear
conv6.2	120×160	3×3,32,stride1	conv7	240×320	3×3,32,stride1
conv6.3	60×80	3×3,32,stride1	conv8	240×320	1×1,2,stride1
conv6.4	60×80	3×3,32,stride1			

**Table 4:** Categories, scenes, and frame indices of testing set.

Category	Sequence	Frame Indices	Sequence	Frame Indices
Pan	continuousPan	600-1149	intermittentPan	1200–2349
	I_MC_01	1–300	I_SM_01	1–300
	I_SM_02	1–300	I_SM_03	1–300
	O_MC_01	1–425		
P&T	O_SM_01	1–425	O_SM_02	1–425
	O_SM_03	1–425		
Zoom	zoomInZoomOut	500-814		

**Table 5:** Average F-Measure on the test videos with different parameters range for the motion map.

Parameters Range			Best F-Measure	Worst F-Measure
θ=20	N∈U	β≤20	0.8135	0.7613
	N>8	β=40	0.8127	0.7978
θ=50	N>8	β≤10	0.8105	0.7624
	N>16	β>10	0.8027	0.7782
θ=80	N>16	β=5	0.7715	0.7385
	N=64	5<β≤20	0.7683	0.7517

**Table 6:** Evaluation values of seven methods on the test dataset. The best results are shown in **bold**.

Method	Pan			P&T			Zoom			Mean		
	P	R	F	P	R	F	P	R	F	P	R	F
PAWCS [5]	0.2221	0.8123	0.3355	0.1413	0.8797	0.2433	0.0395	0.8269	0.0754	0.1835	0.8320	0.2867
SWCD [18]	0.3666	0.5610	0.3786	0.2510	0.5393	0.3416	0.0656	0.3445	0.1101	0.3077	0.5354	0.3441
SCBU [23]	0.7902	0.3094	0.4189	0.8651	0.4438	0.5867	0.7691	0.2191	0.3410	0.8087	0.3378	0.4576
MLBS [24]	0.5818	0.4431	0.4381	**0.8920**	0.5329	0.6671	-	-	-	0.6749	0.4701	0.5068
FgSegNet [12]	0.3702	0.7261	0.4323	0.3719	0.8748	0.5165	0.0416	0.9085	0.0796	0.3408	0.7832	0.4232
ICNET-CDNET [28]	0.0963	0.7017	0.1545	0.1040	0.6809	0.1804	0.0044	0.8090	0.0088	0.0900	0.7058	0.1483
HTFnetSeg	**0.8946**	**0.8162**	**0.8440**	0.6001	**0.9306**	**0.7114**	**0.8925**	**0.9206**	**0.9063**	**0.8141**	**0.8569**	**0.8135**

**Table 7:** Evaluation values of the model if the different components are added. The best results are shown in **bold**.

Method	Precision	Recall	F-Measure
DFFnetSeg [14]	0.5185	**0.9032**	0.5852
HTFnet	0.6899	0.8953	0.7349
+Motion Map	0.8141	0.8569	0.8135
+Fine-tune	0.7497	0.8947	0.7841
+Fine-tune+Motion Map	**0.8707**	0.8570	**0.8501**
